# The Reactive Plasticity of Hippocampal Ionotropic Glutamate Receptors in Animal Epilepsies

**DOI:** 10.3390/ijms20051030

**Published:** 2019-02-27

**Authors:** András Mihály

**Affiliations:** Department of Anatomy, Faculty of Medicine, Szeged University, Kossuth L. Ave. 40., P.O. Box 427, H-6701 Szeged, Hungary; mihaly.andras@med.u-szeged.hu

**Keywords:** AMPA receptor, kainate receptor, NMDA receptor, hippocampus, epilepsy, glutamate, neuronal plasticity

## Abstract

Ionotropic glutamate receptors (iGluRs) mediate the synaptic and metabolic actions of glutamate. These iGluRs are classified within the α-amino-3-hydroxy-5-methyl-4-isoxazole propionic acid (AMPA)-type, kainate-type, and *N*-methyl-d-aspartate (NMDA)-type functional receptor families. The iGluR assemblies are regulated by transcription, alternative splicing, and cytoplasmic post-translational modifications. The iGluR subunit proteins are transported from the endoplasmic reticulum, inserted into the synaptic membranes, and anchored at their action site by different scaffolding and interacting proteins. The functional properties of iGluRs depend on their subunit composition, the amino acid sequence of the protein domains, and the scaffolding proteins in the synaptic membranes. The iGluRs are removed from the membranes by enzymatic action and endocytosis. Hippocampal iGluRs are rearranged through the upregulation and downregulation of the subunits following deafferentation and epileptic seizures. The rearrangement of iGluRs and the alteration of their subunit composition transform neurons into “pathological” cells, determining the further plasticity or pathology of the hippocampal formation. In the present review, we summarize the expression of AMPA, kainate, and NMDA receptor subunits following deafferentation, repeated mild seizures, and status epilepticus. We compare our results to literature descriptions, and draw conclusions as to the reactive plasticity of iGluRs in the hippocampus.

## 1. Introduction

Glutamic acid (Glu) is the main excitatory neurotransmitter in the mammalian brain. Neurons, astrocytes, and probably other glial cells use Glu for information processing [1,2]. Interneuronal synapses use glutamate as a transmitter amino acid and as an extracellularly diffusing neuromodulator targeting both presynaptic and postsynaptic structures [1,2,3]. Glu is released primarily from synaptic vesicles into the synaptic cleft. Glu diffuses fast from the synaptic cleft, partly affecting the receptors of the presynaptic membrane, and partly reaching the receptors of the postsynaptic membrane [3]. In the short term, the activation of ionotropic glutamate receptors (iGluRs) causes membrane depolarization/excitation through cation influx [1,3]. Long-term synaptic plasticity effects include conformational, localizational, qualitative, and quantitative changes of the presynaptic and postsynaptic iGluRs, the anchoring macromolecules, and molecular aggregates [3,4]. The gene expression alterations of the cell nucleus caused by the iGluR signaling will be mediated by synapto-nuclear protein messengers inside the neuron [5]. 

The short-term and long-term synaptic effects of Glu are mediated by ionotropic and metabotropic Glu receptors (iGluRs and mGluRs) [1,3,6]. The iGluRs are cation channels that open when Glu is bound to their extracellular loop [1,3,6]. The mGluRs are not conducting ion fluxes when they bind Glu; instead, they mediate intracellular biochemical processes through G-proteins (which may target ion channels, too) [1,6]. The iGluRs mediate synaptic facilitation and depression, long-term potentiation (LTP), and long-term depression (LTD), which are underlying the cellular processes of learning in the brain [1,7]. The strong and sustaining release of Glu is responsible for excitotoxicity in the brain [8,9]. The excitotoxic effects of Glu are manifested as neuronal shrinkage, mitochondrial vacuolization, neuronal cell death with concomitant astroglial swelling, microglial activation, and the sprouting of microvessels [8,9,10]. There are extensive reviews in the literature discussing the functional significance of the iGluRs in neuronal plasticity [11] and neurotoxic damage [9]. The present review focuses on the iGluR family members, treating the possible alterations of the subunit composition of the ionotropic receptors in animal epilepsies. The author’s own results cited in this review were presented in five in extenso publications [12,13,14,15] and in one congress report [16].

## 2. Types of iGluRs in the Mammalian Brain

**Figure 1:**
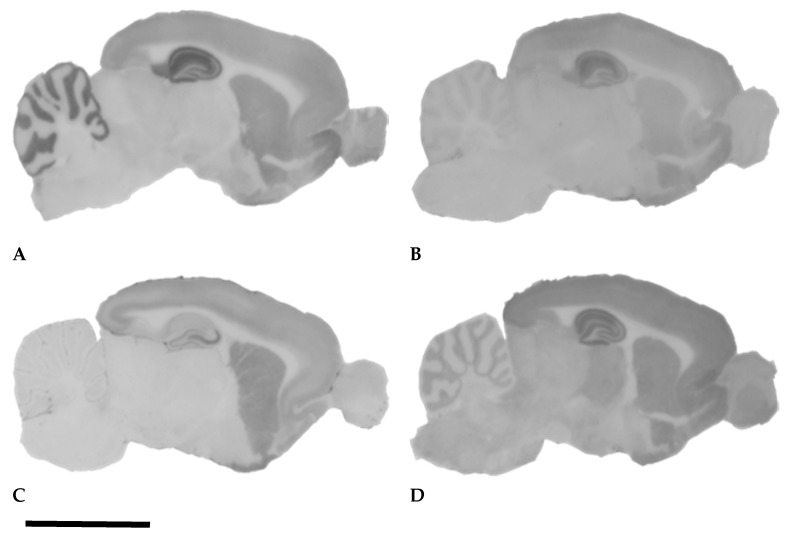
Histoblot images displaying the localization of the α-amino-3-hydroxy-5-methyl-4-isoxazole propionic acid (AMPA) receptor subunits GluA1-4 and GluA2 in the rat brain: GluA1-4 (**A**), GluA2 (**B**); the kainate receptor subunit GluK5 (**C**), and the *N*-methyl-D-aspartate (NMDA) receptor subunit GluN1 (**D**). Note the strong anti-GluA1-4 and anti-GluN1 staining of the dentate molecular layer; the stratum oriens (SO), stratum radiatum (SR), stratum lacunosum (SL), and stratum moleculare (SM) of the Ammon’s horn. The anti-GluK5 serum stains the stratum lucidum (SLUC) of CA3 strongly (**C**). The staining density of the dentate fascia and the SO in CA1 is medium (**C**). The granular and pyramidal layers display weak GluN1 immunoreactivity (**D**). The histoblot signal of the GluA2 subunit (**B**) is similar to that of the GluA1-4, although weaker. We noticed that the CA1 region with anti-GluA2 was labeled stronger than the rest of the Ammon’s horn (**B**) [16]. See Appendix A for methods. Bar: 1 cm.

The excitatory neurotransmission in the central nervous system (CNS) is largely maintained by Glu; therefore, the grey matter of the rodent brain contains high amount of iGluRs (Figure 1). There are three main structurally and pharmacologically different iGluR classes in the adult mammalian brain: α-amino-3-hydroxy-5-methyl-4-isoxazole propionic acid (AMPA) receptors, kainate receptors, and *N*-methyl-D-aspartate (NMDA) receptors [1,3,6]. Apart from these major iGluRs, there are some ill-characterized “delta” type or “orphan” receptors (GluD1 and GluD2), which share structural homology to AMPA and kainate receptors; however, they are not gated by Glu, and do not function as cation channels. Instead, they connect presynaptic and postsynaptic elements with the help of an extracellular glycoprotein [17]. 

The iGluRs were named after their selective pharmacological agonists: AMPA receptors (AMPARs) after the agonist α-amino-3-hydroxy-5-methyl-4-isoxazole propionic acid (AMPA), kainate receptors (KARs) after the agonist kainic acid (KA), which is a toxic glutamate analogue, and NMDA receptors (NMDARs) after the agonist *N*-methyl-D-aspartate (NMDA) [1]. The iGluRs are heterotetramers consisting of four transmembrane proteins with a large extracellular ligand-binding domain, a common pore-forming transmembrane domain, and an intracellular C-terminal domain: these transmembrane proteins are the receptor subunits [1,3,18]. The subunits are structurally different: the peptide sequences of the extracellular domains are frequently used for the generation of antibodies in immunohistochemistry [1]. The transmembrane domains of the four subunits build up the pore of the ion channel [1,6]. The intracellular C-terminal domain is important for connecting the receptor to scaffolding proteins [1,6]. The AMPA receptor has four subunits named GluA1, GluA2, GluA3, and GluA4 [18]. Kainate receptor (KAR) subunits are called GluK1, GluK2, GluK3, GluK4, and GluK5 [18]. The NMDA receptor has seven subunits named GluN1, GluN2A, GluN2B, GluN2C, GluN2D, GluN3A, and GluN3B [18]. The subunits are coded by corresponding genes: the AMPA subunit genes are named GRIA1, GRIA2, GRIA3, and GRIA4, the kainate subunit genes are accordingly GRIK1, GRIK2, GRIK3, GRIK4, and GRIK5, and the NMDA subunit genes are called GRIN1, GRIN2A, GRIN2B, GRIN2C, GRIN2D, GRIN3A, and GRIN3B [18]. The acronyms of the subunit proteins and genes are official terms created by the International Union of Basic and Clinical Pharmacology (IUPHAR) [18]. The receptor tetramers are formed through the assembly of four, mainly different receptor subunit proteins [1,3,6,18]. There are multiple subunit variants due to the alternative splicing and editing of the RNA transcripts; these are listed in more comprehensive reviews [1].

### 2.1. Tissue Localization of iGluRs and Subunits in the Hippocampus

#### 2.1.1. AMPA Receptors and Subunits

Several immunohistochemical studies have described the hippocampal localization of the iGluRs [19,20,21,22,23,24,25]. These studies have described the widespread occurrence of AMPARs in pyramidal neurons [19,20,22] and interneurons [23,24,25]. The early descriptions utilizing immunoperoxidase methods [19,20,22] and subcellular fractionation [26] indicated the postsynaptic localization of the receptor proteins. 

The application of the subunit-specific antibodies to GluA1, GluA2, and GluA3 in histoblotting provided similar immunostaining, and also made the semiquantitative densitometry of the subunit proteins in the histoblots possible [12,13,14]. The histoblotting method omits tissue fixation: the brains are rapidly frozen in isopentane and then sectioned with cryostat; then, the sections are melted onto microscope slides, and the proteins of the sections are transferred to nitrocellulose membranes [12,13]. The detection of the subunit proteins with antibodies is done on the nitrocellulose membranes, which are incubated with the subunit-specific antibodies, and alkaline phosphatase-conjugated secondary antibodies [12,13]. The signal is detected through the visualization of the enzyme activity [12,13]. These histoblots did not give us cellular details, but instead displayed the distribution and density of the receptor subunit protein precisely in the different layers and areas of the hippocampus (Figure 1). Therefore, histoblots were suitable for the semiquantitative detection of the alterations of subunit protein expression in the hippocampus [12,13,14]. The strongest immunostaining has been observed with the pan-AMPA antibody, which is reacting with every subunit of the AMPAR [27,28]; the GluA1 antibodies stain with medium density [12,13,14], whereas the GluA2 antibody results in a weaker signal (Figure 1). The GluA1*_flop_* antibody stains similarly to GluA1, but gives a weaker signal [12]. Antibodies stain mainly the neuropil: the most intense staining is experienced in the stratum oriens (SO), stratum radiatum (SR), and stratum lacunosum (SL) of the CA1 region. The least intense staining is found in the hilus of the dentate fascia and in the SR and stratum lucidum (SLUC) of CA3 [12,13]. Frozen sections from perfusion-fixed mouse brains stained with GluA2/3 antibodies [15] have also stained several multipolar neurons in the hilus of the dentate fascia, in which the neurons were supposedly the hilar mossy cells [15,24,29]. 

#### 2.1.2. Kainate Receptor Subunits

**Figure 2:**
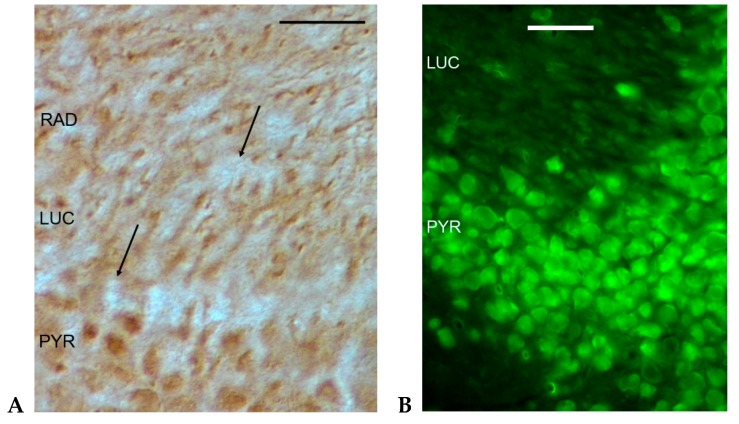
Immunohistochemical localization of GluK1 (**A**) and GluK2 (**B**) antibodies in the CA3 region of the murine hippocampus. The immunohistochemical picture suggests mainly postsynaptic GluK1 and GluK2 localization. PYR: pyramidal layer; LUC: stratum lucidum; RAD: stratum radiatum. Arrows on Figure 2A point to unstained mossy fiber terminals. See Appendix A for methods. Bars: 50 µm.

The application of the histoblotting procedure for the detection of the GluK5 subunit resulted in a homogeneous neuropil staining in SO, SR, stratum lacunosum (SL), and stratum moleculare (SM) in the mouse and rat hippocampus (Figure 1). We detected strikingly strong GluK5 immunostaining in the stratum lucidum (SLUC) of CA3 (Figure 1C). This strong immunostaining of the SLUC in CA3 was characteristic of the histoblotting procedure, and precisely depicted the area where the mossy fibers form giant synapses (Figure 1C). This strong immunostaining of the CA3 SLUC layer has not been detected with GluK2 antibody applied on frozen sections from perfusion fixed mouse brains [15]. The immunohistochemical localization of GluK1 in histological sections of the mouse hippocampus displayed punctate immunostaining of the neuropil in the SR, and immunoreactivity of the CA3 pyramidal cell bodies and dendrites in the SLUC and SR of the CA3 (Figure 2A). The GluK2 antibody stained the cytoplasm of the CA3 neurons in mice [15] (Figure 2B). The strong GluK5 signal originating from the SLUC of the CA3 in histoblots (Figure 1C) suggested precise target localization, because the staining strictly corresponded to the area occupied by the mossy fiber axon terminals [13,15,16]. Indeed, GluK5 and GluK2 were localized in immunohistochemical sections [30] similarly to our histoblots: strong staining of the SLUC was detected in the CA3, which was not seen in the GluK4/5 knockout mice [30]. This strong immunostaining originated from the synaptic KAR content of the mossy fiber CA3 pyramidal cell synapses as observed also with immunoelectron microscopy of KAR-specific scaffolding proteins [30].

#### 2.1.3. NMDA Receptor Subunits

Histoblotting with the anti-GluN1 serum revealed a laminar staining pattern in the hippocampus, which was similar to the AMPAR immunostaining (Figure 1). The most intense neuropil staining has been found in the SO, SR, and SL of the CA1 [12,13,15]. The staining of these layers was slightly increasing toward the subiculum. Strong immunostaining was experienced in the stratum moleculare (SM) of the dentate fascia, whereas weak staining was observed in the hilus, SM, SR, and SLUC of CA3, and in the pyramidal cell and granule cell layers [12,13,15]. The histoblots prepared with anti-GluN2A and anti-GluN2B sera stained the CA1 and the molecular layer of the dentate fascia similarly to the staining of the GluN1 serum, but with weaker signal [12,13]. Light microscopic immunohistochemistry with GluN1, GluN2A, and GluN2B antibodies have mainly stained the synaptic layers of the CA1 and the entire dentate molecular layer [15,21,28]. The localization of GluN3 subunits in the hippocampus also proved the ubiquitous neuronal localization pattern [31]. 

#### 2.1.4. Rearrangement of iGluR Subunits Following Chronic Deafferentation

Destruction of the lateral entorhinal cortex (LEC) with electrocoagulation and suction in rats [12] has caused characteristic alterations of iGluR subunits in the hippocampus mainly on the side of the lesion [12]. Forty days following the ablation of the LEC, the GluA1*_flop_* decreased in the SO, SR, SL, and SM of CA1, whilst GluN1, GluN2B, and GluK5 increased in the SL and SM of the CA1 and SM of the dentate fascia. These were the areas where the excitatory afferents from the LEC terminated, which were degenerated following the ablation [12]. These results highlight the importance of the activity of the afferent presynaptic terminals in the maintenance of the postsynaptic iGluR subunit composition [12]. As to the increase of the GluN2B subunit, the extrasynaptic accumulation of NMDA receptors or receptor subunits has to be taken into account [12,32]. The extrasynaptic NMDARs contain a GluN2B subunit and appear in Huntington disease, ischemia, and epilepsy [32]. These extrasynaptic NMDA receptors increase the neurotoxicity of glutamate [32]. The rearrangement of the hippocampal iGluRs following temporoammonic path afferent degeneration [12] has caused spatial memory deficiency [33] and attenuated the acute hippocampal seizures in the LEC-ablated animals [12].

### 2.2. Electron Microscopic Immunohistochemistry of the iGluR Subunits in Rodent Hippocampus

After the early electron microscopic immunoperoxidase observations, which emphasized the postsynaptic iGluR localization [19,20,21,22], postembedding immunogold electron microscopic studies revealed both presynaptic and postsynaptic localizations of iGluR subunits in the hippocampus [34]. Electron microscopic localization studies postulated the presence of all four AMPAR subunits in type 1 (asymmetric) synapses in the area of the postsynaptic density [34]. This study also suggested the presence of AMPARs in some interneurons of the pyramidal layer and alveus of CA1 [34]. GluA1 and GluA2 subunits have been localized not only postsynaptically, but also in presynaptic axons in hippocampal slice cultures and young rats [35]. Investigations of KAR localization have revealed that GluK2 and GluK4/5 subunits were localized both presynaptically [31,36] and postsynaptically [31,36] in the CA3 mossy fiber synapses. Similar, ultrastructural postsynaptic localization of the GluN1 protein has been described in asymmetric (type 1) CA1 synapses, indicating the presence of NMDARs in the postsynaptic densities of excitatory hippocampal synapses [37]. Other studies have also described labeled postsynaptic densities with the immunoperoxidase method, as well as the localization of the GluN1 C1 splice variant in presynaptic terminals in the subiculum of the hippocampus in young rats and thin myelinated axons of the hippocampal fimbria, which may indicate the axonal transport of the GluN1 C1 subunit protein [21]. The presynaptic presence of iGluRs may refer to their functional significance and regulatory activity in axon terminals: presynaptic NMDARs and KARs may regulate the traffic of synaptic vesicles, and may also indicate the exocytosis and endocytosis and the trafficking of the receptor subunit proteins [4,21,31,36,38,39].

## 3. Functional Alterations, Expression, and Distribution of iGluR Subunits Following Seizures

Convulsions are states of hyperexcitation in the brain tissue. Forebrain epileptic convulsions are mediated mainly by Glu [40]. Brain microdialysis detected a significant increase of tissue glutamate during 4-aminopyridine (4-AP)-induced seizures [41]. The non-competitive antagonist of the AMPA receptor, GYKI 52466, reduced the seizure symptoms and increased the seizure latency [42,43], but it did not prevent the swelling of the hippocampal astrocytes in 4-AP seizures [42]. The pharmacological antagonists of KARs prevented the acute pilocarpine seizure [44]. The combination of NBQX (an AMPAR antagonist) and ifenprodil (an NMDAR antagonist) exerted promising antiepileptic actions in kainate-induced murine epilepsy [45]. Antagonists of NMDAR inhibited the Glu release [46] and seizure symptoms [43,46], as well as prevented the post-ictal neuronal damage [47] and the long-term reactive plasticity of the dentate granule cells [45].

The endogenous downregulation of the iGluRs may protect the neurons and the synapses against the toxic effects of Glu [48]. Neuroprotection in acute epileptic fits against the neurotoxicity of Glu can be achieved by blocking the iGluRs with pharmacological antagonists [42,43,44,45,46,47,48,49]. However, the neuronal damage and degeneration, which develop in the chronic seizure, rearrange the wiring of the brain [45,50]. The loss of the neurons and axon terminal degeneration will change the expression, localization, and molecular composition of iGluRs, as it could be seen in our previous experiments: the destruction of the LEC increased GluN1, GluN2B, and GluK5 in the hippocampus [12]. The neuronal loss in CA1 and CA3 during pilocarpine-induced epilepsy significantly increased GluK2 immunoreactivity [15,16]. The neurodegeneration has functional consequences, too. The neuronal loss in the hippocampus affects not only the wiring but also the functions; learning and memory of the experimental animals will decrease significantly [12,33]. The consequences of the seizures are discussed in two sections:

(1) we discuss the alterations of iGluRs following repeated, mild seizures without hippocampal neuronal degeneration [51,52];
(2) we discuss the alterations of iGluRs following chronic epilepsy and hippocampal neuronal damage [15,53].

### 3.1. Alterations of iGluRs after Repeated, Short Convulsions Caused by 4-AP

The acute seizures precipitated by the systemic injection of 4-aminopyridine (4-AP) cause brain edema and increase of the regional cerebral blood flow (rCBF) [52]. We measured elevated glutamate concentration in the striatum lasting for more than 60 minutes [41]. Electron microscopy revealed a slight shrinkage of the neurons [52] and astrocytic swelling [42,52]. The animals successfully recovered from the seizure [42,51,52], because the systemically injected 4-AP was eliminated from the blood plasma with a 65 to 71-minute half-life [54]. When we injected the rats daily for 12 days, most of the animals suffered mild behavioral motor convulsions every day after the injections [13,14,51]. Following the repeated seizures, the animals displayed characteristic alterations of hippocampal iGluRs: significant decrease of AMPAR density (the density of GluA1-4 subunits) in the dentate fascia (hilus and molecular layer) and the SO of the CA3. At the same time, GluA1 was slightly, but significantly, upregulated in the SL and SM of CA1 and the SR and SLUC of CA3 [13]. The density of GluA2 was dropped significantly in the SO, SL, and SM of CA1 and in the hilus of the dentate fascia [13]. Accordingly, hippocampal slices from convulsing animals displayed a significant increase of in vitro cobalt uptake (indicating the increased calcium permeability of non-NMDA receptor channels [13]) in the SL and SR of CA1 and in the dentate fascia [13]. The histoblotting of GluK5 revealed a significant decrease of the density in SLUC of CA3 [13]. Other areas did not show alterations in GluK5 density [13]. The density values of GluN1 and GluN2B did not change, whilst the density values of GluN2A showed significant increases in every layer of the CA1, CA3, and dentate fascia [13]. The electrophysiological–pharmacological investigations of these hippocampal slices revealed a significant increase in the basic excitability (increased population spike amplitudes) [13]. The antagonistic effects of GYKI 52466 (AMPAR antagonist [13]) were decreased significantly in the convulsing animals, indicating the changes in AMPAR subunit composition [13]. The Q/R-edited GluA2 subunit has a key role in the determination of AMPAR cation permeability, receptor kinetics, and blockade by endogenous polyamines [13]. We think that the observed reduction of GYKI 52466 sensitivity is consistent with the appearance of GluA2-lacking AMPARs [13]. The upregulation of the GluN2A subunit exerted no effects on the pharmacological properties of the NMDARs in our slice experiments [13], as it was proved that GluN2B was more likely to be responsible for the augmentation of Glu-induced excitatory activity in epilepsy [38]. The entorhinal cortex of the convulsing rats displayed similar iGluR subunit alterations and increased excitability [14].

### 3.2. Alterations of iGluRs after Pilocarpine Seizures and Hippocampal Neuronal Degeneration

These experiments were performed in mice, which were systemically injected with pilocarpine and developed status epilepticus (SE) in the next 90 minutes [15,16,53]. The surviving animals were investigated two months after the SE with standard histological and immunohistochemical methods, including the detection of GluA1, GluA2, GluA3, GluK2, and GluN1 subunit proteins in the dorsal hippocampus [15]. The hippocampal layers were evaluated through light microscope densitometry performed on the immunohistochemistry sections [15]. The convulsing animals displayed neuronal degeneration in the hippocampus: the neuronal loss was not uniform, but it was present in every hippocampus, consisting of cell loss in CA1 and CA3, as seen with anti-neuron-specific nuclear protein (anti-NeuN) immunohistochemistry [15]. The following main alterations of iGluRs were noted in the pilocarpine-treated mice. The significant decrease of the AMPAR subunits GluA1, GluA2, and GluA3 was always present in the layers of the dentate fascia and SR and SLUC of the CA3 [15]. Despite this density decrease, the neurons in the pyramidal layer of the Ammon’s horn and neurons in the hilus of the dentate fascia displayed strong immunostaining with the GluA2/3 antibody [15]. This GluA2/3-like immunostaining was also detected in the granule cell layer of the dentate fascia [15]. A significant decrease of AMPARs was detected in the SR of the CA1 region [15]. The significant increase of GluK2 was concomitant in the SR (but not in the SLUC) of the CA3 region [15]. The molecular layer and hilus of the dentate fascia also displayed increased density of immunostaining with GluK2 antibody in the Balb/c mice [15]. The NMRI-strain mice displayed a significant decrease of GluK2 in the hilus of the dentate fascia [15]. The densities of GluN1 either decreased (in Balb/c mice) or did not show alteration (in NMRI mice) [15]. A significant increase of GluN1 immunostaining density was observed in the NMRI mice in the SM and SL of the CA1 [15]. Detectable GluN1 immunostaining was present in the granular layer of the dentate fascia of the control and the epileptic animals [15]. Upon testing these pilocarpine-treated chronically seizing animals for learning and memory with the Barnes maze method [55], significantly worse learning and memory capabilities were measured (unpublished, preliminary results from our laboratory).

## 4. The Reactive Plasticity of iGluRs in Animal Epilepsies

### 4.1. AMPA Receptors

Epileptogenesis is associated with enhanced glutamatergic neurotransmission [56]. Glutamate induces the increase of the cytoplasmic level of free Ca^2+^, which in turn enhances neuronal and astrocytic Glu release [57,58]. The intracellular Ca^2+^ accumulation also triggers molecular cascades involving several intracellular messenger systems, which finally cause the death of the neuron [9,40]. The activation of the Ca^2+^-permeable iGluRs initiates cell damage and neuronal death [6,9,40]. 

The first glutamate receptor with known conductance to Ca^2+^ was the NMDAR [57]. Later, it was shown that some AMPARs are also permeable to Ca^2+^ [59,60]. In AMPARs, the GluA2 subunit is responsible for Ca^2+^ gating: the GluA2 restricts Ca^2+^ permeability [59,60]. The GluA2 hypothesis was set in 1997, stating that Ca^2+^-permeable AMPARs are responsible for cell death in status epilepticus in experimental animals [59], because it was observed that status epilepticus in adult rats caused the downregulation of GluA2 (and GluA3) mRNA [59]. Then, in a second phase of this pathological process, neurons died in the GluA2/3 downregulated area of the hippocampus [59,60]. Region-specific alterations in the phosphorylation of the GluA1 subunit were also detected in rats with pilocarpine epilepsy [61]. Pilocarpine convulsions caused the changes of serine845 and serine 831 phosphorylation of GluA1 in these animals, and it is known that dephosphorylation of the subunit is responsible for the desertion of the AMPAR from the synaptic membranes [61]. A decreased expression of GluA1 and GluA2 mRNAs was detected in the hippocampus during the first week following pilocarpine-induced status epilepticus in rats [62,63]. Electrophysiology has revealed the presence of GluA2-lacking, Ca^2+^-permeable AMPAR in the pyramidal neurons of epileptic animals [64]. The presence of Ca^2+^-permeable AMPARs has also been validated in epileptogenic human hypothalamic hamartomas [65]. These tumorous patients developed characteristic refractory seizures, and the electrophysiological investigation of needle-biopsy tumor tissue specimens proved the presence of Ca^2+^-permeable AMPARs [65]. Moreover, the RNA analysis of the tissue proved that the pathological tissues do not contain adenosine deaminase, which is necessary for the Q/R-editing of GluA2 mRNA [65]. 

Our experiments and the literature data proved that in epilepsy, the adult neuronal AMPARs have a greater probability of losing their GluA2 subunit due to downregulation of the subunit protein and/or the subunit mRNA [13,14,15,59,60,64,65]. Besides our experiments [13,14,15], other studies have also revealed the downregulation of GluA2 [59,60,64,65] and GRIA2 [59] following SE in the cerebral cortex. The molecular mechanisms of the downregulation of the GluA2 subunit in epilepsy are not completely understood. Since the hippocampal AMPAR mRNAs are present not only in the cell body, but also in the dendrites [66], we think that a fast adaptation of the local translational processes may operate in the downregulation [66]. The phosphorylation of AMPAR subunits could be also responsible: the phosphorylation–dephosphorylation cycle is responsible for the intracellular traffic and endocytosis [61,67]. It was shown recently that the phosphorylation of GluA1 alone induces an increase of the Ca^2+^-permeability of AMPARs, which probably takes place with the help of scaffolding proteins [68]. 

### 4.2. Kainate Receptors

#### 4.2.1. Presynaptic KARs

The five subtypes of KAR subunits (GluK1–5) co-assemble in various combinations, probably as heteromers [69,70]. Similarly to AMPA subunits, the mRNAs of KAR subunits are subject to modifications, such as alternative splicing and editing, resulting in a relatively large pharmacological heterogeneity of the KARs [69,70,71,72,73]. Levels of the kainate receptor mRNA in the granule cell layer of the dentate fascia have been decreased significantly in limbic seizures [74]. The presence of KAR mRNA in dentate granule cells predicts the presence of functional KARs in mossy fiber axon terminals. The literature data has supported that the mossy fiber synapses contain abundant presynaptic KARs: in the CA3 of the hippocampus, LTP is a presynaptic phenomenon [72]. These KARs probably contain the GluK2 and GluK3 subunits, which can facilitate the glutamate release through Ca^2+^ influx and facilitate the release of Ca^2+^ from intraaxonal pools [75]. These presynaptic receptors presumably contain the subunit GluK5 as well, since the GluK5 (and GluK4) subunits are key proteins in directing KARs to synapses [76]. The importance of the GluK5 subunit in epileptogenesis was suggested by the histoblots, which proved the significant decrease of the GluK5 density following 4-AP seizures in the SLUC of the CA3, according to the termination area of the mossy fibers [13]. However, the role and presence of presynaptic GluK5 in epileptogenesis needs further investigations. 

The downregulation of the presynaptic kainate receptors in Neto-knockout animals increased network inhibition, suggesting the role of presynaptic, interneuron KARs in hippocampal gamma oscillations [77]. Investigations of GluK2 overexpression and knockout animals have suggested that the presence of hippocampal GluK2 promoted seizure activity [78,79]. The GluK1 mRNA is mainly expressed in the glutamatergic principal cells of the hippocampus, while GluK2 mRNA expression was detected in GABAergic interneurons [80]. Presynaptic GluK2-containing KARs modulate glutamate release not only via ionotropic but also via metabotropic modes [69,70]. The possible epileptogenic effects of GluK2 and GluK5 were proved in hippocampal slices with mossy fiber sprouting in the dentate fascia: pharmacological inhibition of the two subunits significantly reduced the ictal discharges [79]. Similarly, 7-nitro-2,3-dioxo-1,4-dihydroquinoxaline-6-carbonitrile (CNQX) inhibition of KARs prevented high-frequency hippocampal oscillations in animal seizures [81]. The epileptogenic effects of the presynaptic KARs may also manifest through the decrease of inhibition, because kainic acid was reducing GABA release in the hippocampus, and GABAergic interneurons express KARs that regulate their activity [75,79,80]. 

#### 4.2.2. Postsynaptic KARs

It is likely that not only the presynaptic mossy fiber axons but also the postsynaptic densities of the CA3 pyramidal cells contain the necessary KAR subunits [30,36,73]. Postsynaptically, the KARs mediate synaptic transmission as nonselective cation channels, and they may be involved in the regulation of neuronal plasticity, such as LTP, because they are permeable to Ca^2+^ [69,70,71]. Postsynaptic KARs may also be involved in the neurotoxicity of glutamate in the CA3 region of the hippocampus [70,71,73]. The CA3 neurons suffer damage in chronic seizures [15]. This CA3 neurodegeneration [15] might be the aftermath of the postsynaptic Ca^2+^-permeable KARs present in these pyramidal cells, and that of the chronic Glu release from the sprouted/ectopic mossy axons [50,53,71,75,82]. The presence of GluK4-5 subunits and their scaffolding proteins in presynaptic mossy axons and postsynaptic spines have been detected with immunogold methods in the CA3 region [30,36]. According to recent reviews, KARs play an essential role in the dentate fascia in chronic, recurrent seizures, mediating the ictal electrophysiological phenomena coupled to reactive plasticity during mossy fiber sprouting into the inner molecular layer (IML) [50,53,75,82]. The sprouting of the mossy fiber system is targeting not only the IML, the hilus, and the SLUC of CA3, but also the infrapyramidal layer of the CA3 close to the hilus, or the CA4 [53,75,82]. The question arises as to whether KARs were present in the postsynaptic elements, the sprouting mossy fibers, or both in the reorganized CA3, CA4, and dentate fascia [50,53,75,82]. This issue needs further investigation, as having information on the subunit composition of the receptors in the sprouted mossy fibers and in their postsynaptic targets could enable better control of these chronic seizures.

### 4.3. NMDA Receptors

The functional NMDA channels are heteromers consisting of two obligatory GluN1 subunits and the other two associated subunits of the GluN2A-D and GluN3A-B subtypes [1,3,6,48,58]. The obligatory GluN1 subunit binds glycine and d-serine, and is widely expressed in CNS neurons [48]. The GluN2A-D subunits bind Glu and determine the potency of Glu and the Ca^2+^-permeability of the NMDAR [48]. These subunits are coded by separate genes: GRIN1, GRIN2A-D, and GRIN3A-B [1,18,58]. It has been shown recently that mutations of the GRIN2A gene are not rare in epileptic patients [83,84], and that the mutations cause significant functional alterations of the NMDA receptor [84]. Concerning the functional properties of the heteromeric complexes, it is known that the GluN2 subunits are important in channel gating, opening, agonist sensitivity, and deactivation kinetics [1,48,57,58,84]. The GluN2B subunit is also important during synaptogenesis and synaptic plasticity [38,39,48]. The receptors containing GluN2A are located mainly in synaptic densities, whilst the GluN2B subunit is supposed to be present also in extrasynaptic NMDA receptors [6,32,39]. Interestingly, destruction of the LEC (deafferentation of the hippocampus) increased the amount of GluN2B subunit in the deafferented CA1 and dentate fascia, probably indicating the presence of non-synaptic NMDA receptors containing GluN2B receptor subunits [12,32]. 

Animal experiments have proven the reactive plasticity of NMDAR subunits in hippocampal epileptogenesis [13,15,85]. Experimental status epilepticus increased GluN1 containing synapses in the dentate fascia [85]. Hypoxic seizures in young rats significantly elevated the hippocampal expression of GluN3 two to four days after the convulsions [86]. Pilocarpine-induced status epilepticus has been found to induce a significant increase of NMDA receptors in dentate granule cells and CA3 pyramidal neurons [38]. The subunit pharmacology experiments have revealed that the GluN2B subunit was responsible for the augmentation of the excitatory activity in these animals [38]. The increase of the GluN2B/GluN2A ratio was also detected in epileptic human brain samples [87]. The phosphorylation of the GluN2 subunit was significantly elevated in animal epilepsy, indicating the increase of the turnover of the subunits [88]. Pentylenetetrazole-induced status epilepticus significantly increased GluN1 and GluN2A total RNA levels isolated from the hippocampus one day after the convulsive event [89]. On the other hand, seven days following pilocarpine convulsions, GluN1 and GluN2A RNAs were significantly downregulated, in comparison to the controls [63]. Seizures induced by intrahippocampal kainic acid first (three days after kainic acid injection) decreased, and then elevated the GluN1 expression in the dentate fascia [90]. An increase of GluN1-like immunoreactivity has been detected in the neurons of the rat hippocampus shortly after the SE, indicating that neurons regulate the receptor traffic according to the synaptic activity [85]. 

**Figure 3:**
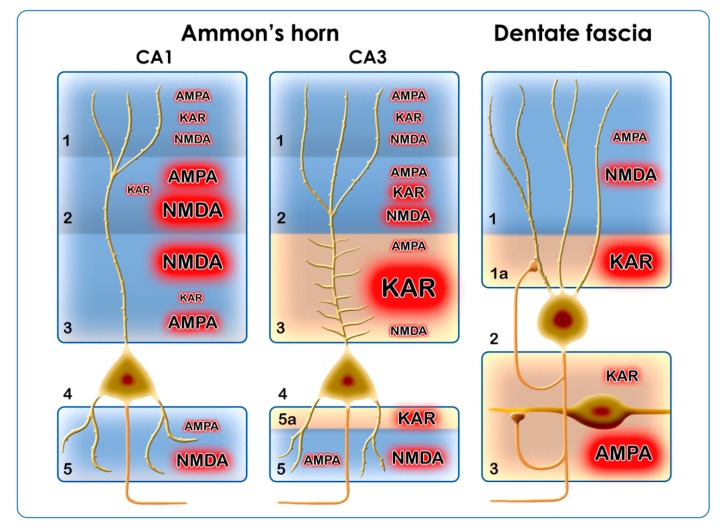
Pictorial summary of the functional significance of hippocampal ionotropic glutamate receptors (iGluRs) in epilepsy. Two regions of the Ammon’s horn (CA1 and CA3) and the dentate fascia are depicted. The pathogenetic importance of the α-amino-3-hydroxy-5-methyl-4-isoxazole propionic acid (AMPA), kainate receptor (KAR), and *N*-methyl-D-aspartate (NMDA) subunit assemblies is shown through the letter size and the red glow around them. Layers in CA1: (1) stratum moleculare (SM); (2) stratum lacunosum (SL); (3) stratum radiatum (SR); (4) stratum pyramidale; (5) stratum oriens (SO) [92]. Layers in CA3: (1) stratum moleculare (SM); (2) stratum radiatum (SR); (3) stratum lucidum (SLUC, modified after [92]); (4) stratum pyramidale; (5) stratum oriens (SO); (5a) infrapyramidal layer containing sprouted mossy axons (modified after [92]). Layers in the dentate fascia: (1) stratum moleculare (SM); (1a) inner molecular layer (IML), where sprouted mossy axons are present; (2) stratum granulosum; (3) hilus of the dentate fascia, where sprouting is present, too [50].

Hippocampal regions display different vulnerability in epilepsy [91]. The CA1 region is among the hippocampal areas where extensive neuronal death was detected in epileptic patients [91]. The damage of the CA1 area may begin with the lasting activation of postsynaptic Ca^2+^-permeable NMDARs [3,9]. The main excitatory afferents of the CA1 originate from the entorhinal cortex (perforant path) and from the CA3 pyramidal cells (Schaffer collaterals) [92]. These excitatory synapses utilize presynaptic and postsynaptic NMDARs [93]. The literature data has proved that the subunit composition of NMDA receptors largely depends on the presynaptic activity [93]; therefore, the synaptic layers display different aggregates of NMDAR subunits [93]. Since the epileptic electrophysiological processes can be inhibited by NMDA antagonists [94], we think that the synapses in the SR and SL develop reactive plasticity in seizure conditions: the Ca^2+^-permeable NMDARs and AMPARs will be segregated in these layers during pathological conditions (Figure 3). High-frequency stimulation and epileptic activity have long-term impacts on these subunit assemblies: the expression [13,15,63], the trafficking [38,85], and the scaffolding molecules [95] are equally affected. The strong excitatory input on the proximal dendritic arborization of the pyramidal cells will maintain the remodeling of NMDARs [13,15,93]. 

We can conclude that the NMDARs are regulated differentially during convulsions: they are upregulated [89] in the acute phase and downregulated later, in the chronic phase of the disease [63], although the dynamics of the changes depended on the type of the experiment [85,86,89]. It is not known if the chronic downregulation [63] was just reflecting the neuronal degeneration, i.e., fewer neurons contain fewer NMDA receptors. The high frequency stimuli increased the amount of GluN1, GluN2A, and GluN2B subunits [86,88,89] by influencing the translation and the trafficking [85,95]. We do not know to what extent these subunit alterations are involving the presynaptic and the postsynaptic NMDARs: both NMDARs play important roles in long-term alterations [58] and probably in neurotoxicity, too. Although at this time it is not possible to draw firm conclusions as to the pathogenetic role of the GluN2A and GluN2B subunits in human epilepsy, we have to note that clinical observations with positron emission tomography have indicated the significant increase of NMDA receptor ligand binding in human focal epilepsies [96]. Although the increase of ligand binding [96] could have been the consequence of the appearance of extrasynaptic or non-neuronal NMDARs [32], these firm observations on animal and human tissues point toward the crucial significance of NMDARs in epilepsy. 

## 5. Conclusions

- Summarizing our own results and the literature data, we can conclude that the main alteration of AMPARs during epilepsy is the increase of their Ca^2+^ permeability. This will be achieved through the downregulation of the GluA2 subunit at the transcriptional, translational, and/or post-translational levels. The phosphorylation/dephosphorylation cycles of the subunits are important too, because phosphorylation alone induces Ca^2+^ permeability and changes the trafficking of the receptor. AMPARs are localized in every region of the hippocampal formation; therefore, the transformation of AMPARs will affect every region and cell type of the hippocampus (Figure 3).
- The Ca^2+^ permeability of KARs contributes to epileptogenesis presynaptically by increasing the release of Glu, and postsynaptically by increasing intracytoplasmic Ca^2+^ concentration and the neurotoxicity of Glu. The KARs have an outstanding role in the DF–CA4–CA3 regions. In chronic pilocarpine epilepsy, there is an extensive axonal sprouting in these regions, which originates from the dentate granule cells. Sprouting mossy fibers in the IML, SLUC, and hilus of the dentate fascia form synapses [97] and probably maintain neurotoxicity through their KAR content (Figure 3).
- The NMDARs display reactive plasticity in epilepsy; their localization and subunit composition are subject to changes from the acute phase until the chronic phase of epilepsy. The alterations of the GluN1 and GluN2 subunits include transcriptional, translational, post-translational, local phosphorylation, and trafficking changes. According to literature data, the GluN2A and GluN2B subunits are the most frequently involved. The NMDARs function mainly in the postsynaptic regions of the CA1 and the ML of the DF, where they may display synaptic and extrasynaptic localizations. In every case, they mediate the toxic effects of Glu (Figure 3).
- The reactive plasticity of the iGluRs in different hippocampal regions accommodates to neuronal types (principal neurons/interneurons) and afferent connections (Figure 3). The inhibitory interneurons of the hippocampus utilize KARs, NMDARs, and Ca^2+^-permeable AMPA receptors, and these iGluR combinations may be responsible for the ongoing degeneration of hippocampal inhibitory interneuron populations in epilepsy [98].

## Abbreviations

AMPAR	α-amino-3-hydroxy-5-methyl-4-isoxazole-propionate receptor
NMDAR	*N*-methyl-D-aspartate receptor
KAR	kainate receptor
iGluR	ionotropic glutamate receptor
Glu	glutamic acid
GYKI 52466	4-(8-methyl-9*H*-[1,3]dioxolo[4,5-h][2,3]benzodiazepin-5-yl)aniline, AMPAR antagonist
CNQX	7-nitro-2,3-dioxo-1,4-dihydroquinoxaline-6-carbonitrile, KAR antagonist
NBQX	2,3-dihydroxy-6-nitro-7-sulfamoyl-benzol[f]quinoxaline, AMPAR antagonist
4-AP	4-aminopyridine
CA1, CA2, CA3, CA4	Cornu Ammonis regions in the hippocampal formation
DF	dentate fascia
SO	stratum oriens
SR	stratum radiatum
SLUC	stratum lucidum
SL	stratum lacunosum
SM	stratum moleculare
SE	status epilepticus
LTP	long-term potentiation
LTD	long-term depression
NeuN	neuron-specific nuclear protein
LEC	lateral entorhinal cortex
rCBF	regional cerebral blood flow
NMRI-strain mice	NMRI: abbreviated from Naval Medical Research Institute (inbred albino mouse line)
Q/R editing	RNA editing at the Q/R site: adenosine is converted into inosine by oxidative deamination. Q/R editing leads to the exchange glutamine to arginine during translation

## Appendix A

The procedure of histoblotting was described in detail previously [12,13,14,27]. The antibodies used in histoblots depicted in Figure 1 are as follows: the pan-AMPA (GluA1-4) antibody was raised in rabbit against a fusion peptide which has a sequence homology in all AMPA receptor subunits [27,28]. The pan-AMPA antibody was a gift from Elek Molnar (Bristol University). The anti-GluA2 serum (diluted to 1:200) manufactured by Chemicon, the monoclonal anti-GluN1 serum (diluted to 1:200) by BD PharMingen and the rabbit anti-GluK5 serum (diluted to 1:200) made by Abcam. The mouse tissues on Figure 2A,B were perfusion-fixed, sectioned at 25 µm thickness and reacted with anti-GluK1 and anti-GluK2 sera manufactured by Alomone Labs. The antibody was diluted to 1:200 and the binding was visualized with the streptavidin-peroxidase system (Figure 2A) and secondary antibodies labeled with Alexa Fluor 488 (Molecular Probes) (Figure 2B). The shaping of hippocampal neurons and the layers’ nomenclature of Figure 3, and in other parts of the text were adopted from Stephan [92].
